# Longitudinal Evaluation of Multi-phasic, Odontological and Nutritional Associations in Dentists (LEMONADE Study): Study Design and Profile of Nationwide Cohort Participants at Baseline

**DOI:** 10.2188/jea.JE20070458

**Published:** 2009-03-19

**Authors:** Kenji Wakai, Mariko Naito, Toru Naito, Haruo Nakagaki, Osami Umemura, Makoto Yokota, Nobuhiro Hanada, Takashi Kawamura

**Affiliations:** 1 Department of Preventive Medicine/Biostatistics and Medical Decision Making, Nagoya University Graduate School of Medicine, Nagoya, Japan; 2 Section of General Dentistry, Department of General Dentistry, Fukuoka Dental College, Fukuoka, Japan; 3 Department of Preventive Dentistry and Dental Public Health, School of Dentistry, Aichi-Gakuin University, Nagoya, Japan; 4 Department of Dentistry, Aichi San-no-maru Hospital, Nagoya, Japan; 5 Department of Periodontology and Endodontology, Kyushu Dental College, Fukuoka, Japan; 6 Department of Oral Health, National Institute of Public Health, Ministry of Health, Labour and Welfare, Wako, Saitama, Japan*; 7 Kyoto University Health Service, Kyoto, Japan

**Keywords:** oral health, general well-being, diet, cohort studies, dentists

## Abstract

**Background:**

To examine the association between oral health and general well-being, we are currently conducting a nationwide cohort study comprising members of the Japan Dental Association (JDA). Herein, we describe the study design and the profile of the participants at baseline.

**Methods:**

From 2001 through 2006, the participants completed a baseline questionnaire that surveyed factors related to lifestyle, general health, and oral health. Morbidity and mortality have been monitored by using information from fraternal insurance programs operated by prefectural dental associations. All respondents provided written, informed consent for participation and the use of their insurance data.

**Results:**

A total of 21 272 JDA members participated in the baseline survey (response rate, 36.2%). Their mean age ± SD was 52.3 ± 12.3 years; 8.0% were women. Among the respondents, 30.2% of men and 10.7% of women were current smokers; 73.5% of men and 44.8% of women were current drinkers. The cohort scored higher on oral health indices than did the general Japanese population: dentists were more likely to brush their teeth ≥3 times/day, to have ≥20 teeth, to have fewer lost teeth, to be free from periodontal diseases, and to have higher General Oral Health Assessment Index scores. There was, however, considerable inter-individual variation in scores on the indices.

**Conclusions:**

More than one-third of JDA members participated in the study. Their oral average health status was better than that of the general population. Nevertheless, it will be possible to compare morbidity and mortality between those with better and worse scores on oral health indices.

## INTRODUCTION

Oral health is now known to be related to systemic diseases.^1^ Tooth loss may result in an unbalanced diet,^2^^–^^4^ which in turn may lead to chronic diseases such as cardiovascular disease and cancer. Periodontal infections may adversely affect glucose tolerance and accelerate hyperlipidemia^5^ and atherosclerosis.^6^ These effects may be mediated by endotoxins, such as lipopolysaccharide, and/or inflammatory cytokines, such as tumor necrosis factor-alpha, interleukin-1 beta, and interleukin-6.^5^^,^^7^ Poor oral health has also recently been associated with total mortality.^8^^,^^9^

The associations of periodontal disease and tooth loss with the incidence of systemic diseases, particularly cardiovascular disease^8^^,^^10^ and cancers,^8^^,^^11^ and with total mortality^8^^,^^9^ have been examined in case-control and cohort studies, but the results were inconsistent. Cohort studies are superior to case-control studies because they avoid recall and selection biases. A prospective study of the relation between oral health and systemic diseases or premature death in the general population would be quite costly, as it would require a considerable number of participants, individual oral examinations by dentists or dental hygienists, and painstaking long-term follow-up for target diseases or death.

We thus attempted to conduct a nationwide prospective study among Japanese dentists to examine the association of oral health with total mortality and systemic diseases, including cardiovascular disease, cancer, and other diseases. Because the participants were dentists, information on oral status could be obtained by means of a questionnaire survey. In addition, information on mortal and morbid events could be collected easily from fraternal insurance organizations operated by the participants’ affiliated dental associations. Moreover, we assumed that dentists would be more interested than the general population in the association between oral and general health. Similar cohorts of physicians,^12^ nurses,^13^ and various healthcare professionals including dentists^13^^–^^15^ have provided valuable information. In this article, we describe the study design and the profile of cohort participants at baseline, including information on demographic characteristics, lifestyle, general health status, and oral health. Our study was designated the Longitudinal Evaluation of Multi-phasic, Odontological and Nutritional Associations in Dentists (LEMONADE) and the participants are now followed for outcome events.

## METHODS

### Participants and baseline survey

The participants of the present study were members of 46 prefectural dental associations affiliated with the Japan Dental Association (JDA). The JDA is a unique dental professional organization that principally comprises dental practitioners; it had enrolled 67.2% of all dentists in Japan at the end of 2006. The study participants completed a baseline questionnaire distributed by prefectural dental associations and were registered from February 2001 through July 2006. The questionnaire was used to collect information on demographic factors, including sex, year of birth, age, marital status, and number of members in household; on occupational environment, including length of career as a dentist, employment (independent or employed), number of holidays per month, working hours per week, and number of employees (if any); on physiological status, including height, weight, and usual blood pressure (participants were not instructed to re-measure for the survey); on current history of and medication for chronic diseases including stroke (cerebral infarction, cerebral hemorrhage, and subarachnoid hemorrhage), myocardial infarction, hypertension, diabetes mellitus, hyperlipidemia, and cancer; on family history of cardiovascular diseases, hypertension, diabetes, and cancer; on lifestyle factors including smoking and alcohol drinking habits, sleeping hours, physical activity, consumption of soft drinks, number of breakfasts per week, and dietary intake; on subjective health status including health-related quality of life (QOL), on psychological distress, as measured with the 12-item General Health Questionnaire (GHQ-12)^16^; and on indicators of oral health.

The section of the questionnaire on diet inquired about the intake frequency of 97 common Japanese foods and dishes during the one month preceding the baseline survey. This food frequency questionnaire was designed to estimate daily food group consumption and nutrient intakes, and has been validated.^17^^,^^18^ The oral health factors included in the questionnaire included oral hygienic routines, such as frequency of brushing, flossing, and scaling; number of teeth lost (excluding third molars); use of dentures; dental examination chart; periodontal status classified according to the criteria of the Community Periodontal Index (CPI)^19^^,^^20^; history of periodontal diseases with pockets ≥4 mm; and oral health-related QOL, as determined by using the Japanese version of the General Oral Health Assessment Index (GOHAI).

The dental examination chart was recorded in the participant’s office. Participants were asked to assess their periodontal status and to classify it according to the CPI criteria, which were described in the questionnaire. Periodontal status was assessed for 6 teeth in the dentition, as specified in the WHO protocol for CPI measurement.^19^ Each segment was then designated as healthy (score 0), bleeding but without dental calculus (score 1), with calculus but without pockets (score 2), with pockets 4.0 to 5.9 mm in depth (score 3), or with pockets ≥6.0 mm in depth (score 4), according to the highest score for an index teeth. The highest score among 6 segments was regarded as the CPI score of the participant. In 41 of the 46 prefectural dental associations, the profession of the individual who assessed the periodontal status (dental hygienist, another dentist, the participant, or other) was also recorded on the questionnaire.

The Japanese version of the GOHAI questionnaire has been adapted to and validated in the Japanese population,^21^ and the national norm for Japanese is obtainable through iHOPE International (Tokyo and Kyoto). GOHAI is a 12-item index summarized in 3 dimensions: (1) physical function, which includes eating, speech, and swallowing, (2) psychosocial function, which includes anxiety or concerns about oral health, self-image, and self-consciousness about oral health, and (3) avoidance of social contacts because of oral problems, and pain or discomfort. Each item was scored from 1 to 5 and the scores were then summed for all items. The total score, therefore, ranges from a minimum of 12 to a maximum of 60. A higher total score indicates better oral health-related QOL.

### Validation study of self-reported periodontal status

To validate the assessment of periodontal status reported on the self-administered questionnaire, we compared the CPI score reported on the questionnaire with the CPI score measured in an oral examination by one of the authors (TN). In this validation study, the section of the questionnaire concerned with dental health was mailed to 288 dentists randomly selected from 953 members of the Fukuoka City Dental Association from April through August 2008. One of the authors (TN) conducted all oral examinations, including CPI measurement, at the clinics of dentists who consented to undergo an examination.

### Follow-up

The study participants are currently under observation for mortality and morbidity by their affiliated prefectural dental associations. They are to be followed until at least March 2009 (March 2010 in some late-starting prefectures) and, if permitted, the follow-up period will be extended. The prefectural dental associations operate their own fraternal insurance programs. When a JDA member dies or suffers a disease, the member or his/her proxy submits a death or medical certificate to claim the insurance benefit through the office of his/her prefectural dental association. Most events requiring hospital admission are recorded as part of this claims process. Mortal and morbid events in study participants are reported to the study office and coded according to the International Statistical Classification of Diseases and Related Health Problems, 10th revision (ICD-10). The cause of death is determined based on the death certificate. If a study participant resigns from their dental association, it is reported and treated as a loss to follow-up.

### Ethical issues

All eligible respondents, ie, those who consented to participation in the study and the use of their insurance data, submitted consent forms to their prefectural dental association office. To assure strict confidentiality, the questionnaire was anonymized with an ID number identical to that used on the consent form. The outcome data of participants are also anonymized at the prefectural dental association offices using the same ID number and forwarded to the study office. The ethics committees of Nagoya University School of Medicine and Aichi Cancer Center (former affiliation of the principal investigator, [KW]) both approved the protocol of this cohort study (Nos. 33 and 8–21, respectively). All participants in the validation study of self-reported periodontal status provided written informed consent. The protocol of this validation study was approved by the ethics committee of Fukuoka Dental College (No. 77), where the study was conducted.

### Statistical analysis

We tabulated the proportions, means, and/ or medians of respondents for selected baseline characteristics by sex and 10-year age subgroup. Body mass index (BMI) was calculated by dividing self-reported body weight (kg) by the square of the participant’s height (m^2^). Those who consumed alcohol at least once a month were classified as drinkers. Hypertension was defined as a self-reported systolic blood pressure of ≥140 mm Hg, a diastolic blood pressure of ≥90 mm Hg, or a prescription for medication to treat hypertension. Stroke referred to cerebral infarction, cerebral hemorrhage, and subarachnoid hemorrhage, and coronary heart disease referred to myocardial infarction and angina pectoris. The GOHAI questions were asked in 20 of the 46 prefectural dental associations.

In the validation study of self-reported periodontal status, the CPI score reported on the questionnaire was compared to that measured at an oral examination. We evaluated the consistency between the two sets of assessment of periodontal status by calculating the kappa statistic. In addition, we categorized the participants as those with or without a CPI score of ≥3, and computed sensitivity and specificity. A CPI score of ≥3 on an oral examination by one of the authors (TN) was defined as the reference, or “gold standard.” The exact 95% confidence interval (CI) for sensitivity and specificity was estimated based on the binomial distribution. All the analyses were performed with the Statistical Analysis System version 9.1 (SAS Institute Inc., Cary, NC, USA).^22^

## RESULTS

The questionnaire was delivered to 58 792 JDA members, 22 415 of whom returned it to the study office. We excluded 1074 respondents who did not submit the consent form to the prefectural dental association offices and 69 with missing information on age or sex, leaving 21 272 participants (36.2% of those receiving the questionnaire). The age distribution of the cohort peaked at 40 to 49 years (Table 1), with a mean age ± SD of 52.3 ± 12.3 years (range, 26–98 years). Women were 8.0% of the population (*n* = 1700). As of June 2007, 154 participants have resigned from their dental associations.

**Table 1.** Sex and age distribution of study participants at baseline

Age	Men		Women	
	
	*n*	%	*n*	%
20–29	41	0.2	18	1.1
30–39	2455	12.5	210	12.4
40–49	6713	34.3	599	35.2
50–59	5695	29.1	467	27.5
60–69	2524	12.9	149	8.8
70–79	1572	8.0	178	10.5
≥80	572	2.9	79	4.6

Total	19 572	100.0	1700	100.0

The proportion of participants without a spouse at baseline was higher among women than men (Table 2). More than 90% of men and 75% of women were independent operators of their dental offices. The mean length of career as a dentist was 26.4 years (SD, 12.2) in men and 26.5 years (SD, 13.3) in women.

**Table 2.** Selected baseline demographic, lifestyle, and medical characteristics of participants by sex and age

	Age at baseline							Total
	
	20–29	30–39	40–49	50–59	60–69	70–79	≥80	
Men								
Marital status								
Unmarried (%)	61.0	14.1	3.3	1.3	0.4	0.1	0.2	3.5
Married (%)	39.0	83.8	94.4	96.1	96.9	92.4	82.6	93.2
Widowed or divorced (%)	0.0	2.0	2.3	2.7	2.8	7.5	17.3	3.3
Employment								
Independent (%)	63.4	86.2	94.5	96.9	94.3	82.0	57.9	92.0
Employed by relatives (%)	26.8	10.5	4.0	1.7	2.1	6.4	7.0	4.2
Employed by others (%)	9.8	2.9	1.2	0.9	0.8	0.5	0.2	1.2
Not at work as a dentist (%)	0.0	0.0	0.0	0.1	2.2	9.6	33.7	2.1
Others (%)	0.0	0.4	0.3	0.3	0.6	1.6	1.3	0.5
Years practicing as a dentist (mean ± SD)	4.1 ± 1.4	10.8 ± 2.8	19.0 ± 3.6	27.3 ± 4.1	37.4 ± 4.6	48.3 ± 5.4	56.7 ± 6.7	26.4 ± 12.2
Smoking								
Current smokers (%)	39.0	39.8	32.3	29.7	25.1	21.3	14.5	30.2
Ex-smokers (%)	4.9	22.4	32.7	40.5	43.0	52.6	56.6	37.2
Alcohol drinking								
Current drinkers (%)	70.7	73.0	77.2	76.6	71.6	59.7	45.1	73.5
Ex-drinkers (%)	2.4	2.1	2.3	2.6	5.3	9.7	12.3	3.6
Body mass index ≥25.0 (%)	34.2	33.6	33.2	31.2	24.8	20.9	14.1	30.1
Hypertension (%)^*^	24.3	23.8	31.3	43.7	56.6	66.3	66.8	41.1
History of								
Stroke (%)	2.4	0.5	1.0	1.8	5.1	11.1	12.6	2.9
Coronary heart disease (%)	0.0	0.5	1.2	3.1	7.5	13.2	15.4	3.8
Diabetes (%)	0.0	1.4	3.8	9.3	15.5	17.5	13.8	8.0
Cancer (%)	0.0	0.3	1.0	2.6	6.9	13.7	17.0	3.6
GHQ-12^†^ score [median (interquartile range)]	1 (0–4)	2 (1–4)	2 (0–4)	1 (0–3)	1 (0–3)	1 (0–3)	1 (0–3)	1 (0–4)
(n)^‡^	(41)	(2408)	(6591)	(5548)	(2417)	(1427)	(468)	(18 900)

Women								
Marital status								
Unmarried (%)	72.2	34.1	22.4	14.4	8.1	14.1	5.1	19.2
Married (%)	27.8	55.8	62.8	67.1	67.1	45.2	29.1	59.7
Widowed or divorced (%)	0.0	10.1	14.8	18.5	24.8	40.7	65.8	21.1
Employment								
Independent (%)	44.4	75.2	80.4	80.3	78.5	67.2	57.7	76.8
Employed by relatives (%)	33.3	21.4	16.6	15.2	15.4	13.6	10.3	16.3
Employed by others (%)	16.7	2.9	1.7	1.7	0.7	0.0	0.0	1.7
Not at work as a dentist (%)	0.0	0.5	0.5	1.5	4.0	18.6	32.1	4.4
Others (%)	5.6	0.0	0.8	1.3	1.3	0.6	0.0	0.9
Years practicing as a dentist (mean ± SD)	4.6 ± 0.8	11.5 ± 2.7	19.2 ± 3.8	26.7 ± 5.0	37.1 ± 5.1	49.8 ± 5.9	55.7 ± 7.9	26.5 ± 13.3
Smoking								
Current smokers (%)	16.7	12.0	13.7	10.0	8.1	5.2	3.9	10.7
Ex-smokers (%)	5.6	9.1	10.0	10.0	4.7	9.2	5.2	9.1
Alcohol drinking								
Current drinkers (%)	55.6	51.4	53.9	45.8	34.7	24.4	13.2	44.8
Ex-drinkers (%)	0.0	4.8	3.5	1.7	2.0	1.1	1.3	2.7
Body mass index ≥25.0 (%)	11.1	3.9	10.3	15.7	18.2	19.1	19.5	13.1
Hypertension (%)^*^	5.9	6.3	12.5	26.9	38.3	60.0	77.3	26.1
History of								
Stroke (%)	0.0	0.0	0.3	1.3	0.7	6.2	7.6	1.5
Coronary heart disease (%)	0.0	0.0	0.5	0.6	4.7	11.8	7.6	2.4
Diabetes (%)	0.0	0.0	1.2	1.7	7.4	7.3	6.3	2.6
Cancer (%)	0.0	0.5	4.2	5.1	7.4	11.8	7.6	5.2
GHQ-12^†^ score [median (interquartile range)]	3.5 (1–6)	2 (0–4)	2 (0–4)	2 (0–4)	1 (0–4)	1 (0–3)	1 (0–3)	1.5 (0–4)
(n)^‡^	(18)	(209)	(579)	(455)	(146)	(160)	(61)	(1628)

*Self-reported systolic blood pressure ≥140 mm Hg, diastolic blood pressure ≥90 mm Hg, or taking medication for hypertension.

^†^GHQ-12: 12-item General Health Questionnaire.

^‡^Number of participants with complete data.

The proportion of current smokers was higher among younger men as compared with elderly men: 39.0% of men in their third decade of life smoked, as did 39.8% in their fourth decade and 14.5% in their ninth or tenth decade. However, male ex-smokers were more common in older age groups (Table 2). Approximately 10% of women smoked at baseline, and the trend decreased with age. At baseline, more than 70% of men and more than 40% of women were alcohol drinkers, but the prevalence of former drinkers was higher among the elderly in men. Obesity, defined as BMI ≥25.0 kg/m^2^, was inversely associated with age among men; however, the association was positive among women. The prevalence of hypertension was clearly age-dependent in both sexes. Histories of stroke, coronary heart disease, diabetes, and cancer were also more prevalent among elderly participants and, with the exception of cancer, were more common in men than in women.

With regard to oral health indicators (Table 3), more than half of men brushed their teeth at least 3 times per day, except for elderly men; women brushed their teeth more frequently. Tooth loss markedly increased with age, and the mean numbers of lost teeth were very similar between men and women in the same age categories. Interestingly, inter-individual variations, as shown in the SD values, were very large. The proportions of men and women with at least 20 teeth were greater than 95% for those under the age of 60 years, but dropped quickly as age increased, reaching 22.6% and 21.5% in men and women aged at least 80 years, respectively. The age-specific percentages of those with ≥20 present teeth differed little between sexes. The prevalence of periodontal disease with a CPI score of ≥3 also increased with age in men and women, and was approximately 10% overall. A history of periodontal diseases with pockets ≥4 mm was more common in older participants. Some inconsistency was observed between the proportion of participants with a CPI score of ≥3 and the percentage of those with a history of periodontal disease with pockets ≥4 mm: the former was higher than the latter in men and women aged 20 to 29 years. The proportions, based on the response to the questionnaire, are shown in Table 3 without correction. An inverse correlation was found between age and GOHAI score, which implies that oral health QOL is poorer in elderly participants.

**Table 3.** Selected baseline oral health indices of participants by sex and age

	Age at baseline							Total
	
	20–29	30–39	40–49	50–59	60–69	70–79	≥80	
Men								
Brushing ≥3 times/day (%)	51.2	56.9	56.3	55.7	55.0	45.4	36.6	54.6
Filled teeth (mean number ± SD)^*^	7.9 ± 8.2	10.1 ± 6.4	10.5 ± 6.3	8.4 ± 6.0	5.2 ± 5.4	3.8 ± 5.1	2.8 ± 4.7	8.4 ± 6.5
Tooth loss (mean number ± SD)	1.0 ± 4.4	0.7 ± 1.9	1.2 ± 2.2	2.4 ± 3.7	5.3 ± 7.0	13.0 ± 10.0	18.6 ± 9.6	3.4 ± 6.4
Number of present teeth ≥20 (%)	97.6	99.6	99.3	95.5	80.7	44.4	22.6	89.3
Use of bridges (%)^*^	0.0	10.6	22.1	28.2	23.1	19.8	17.6	22.2
Use of partial dentures (%)	2.4	4.6	11.8	20.8	36.8	55.6	50.3	21.4
Use of full dentures (%)	0.0	0.0	0.0	0.9	7.0	30.2	53.6	5.1
CPI score ≥3 (%)^†^	7.7	5.5	7.8	12.9	19.7	15.8	10.0	10.9
CPI score =3 (%)	5.1	4.7	6.5	9.8	15.1	11.8	7.9	8.6
CPI score =4 (%)	2.6	0.8	1.3	3.1	4.6	4.0	2.0	2.3
No index teeth for CPI measurement (%)	0.0	0.0	0.0	0.4	4.7	23.8	47.5	3.3
History of periodontal diseases with pockets (≥4 mm) (%)^*^	0.0	10.0	13.4	24.1	39.2	51.1	42.5	23.0
GOHAI score (mean ± SD)^‡^	57.8 ± 3.7	56.0 ± 5.3	55.5 ± 5.4	54.5 ± 6.1	53.3 ± 6.9	51.5 ± 7.7	50.5 ± 8.3	54.5 ± 6.3
(Number of participants with sufficient data)	(19)	(1122)	(3158)	(2675)	(1265)	(772)	(241)	(9252)

Women								
Brushing ≥3 times/day (%)	83.3	73.8	72.8	78.9	60.3	59.3	47.9	71.2
Filled teeth (mean number ± SD)^*^	8.3 ± 5.5	11.5 ± 6.3	12.1 ± 5.9	10.8 ± 5.5	7.8 ± 5.1	4.7 ± 5.3	3.6 ± 5.8	10.0 ± 6.3
Tooth loss (mean number ± SD)	1.0 ± 1.7	0.9 ± 1.6	1.4 ± 2.1	2.0 ± 2.5	5.4 ± 6.6	13.1 ± 10.0	19.3 ± 9.6	3.9 ± 6.8
Number of present teeth ≥20 (%)	100.0	99.5	99.5	97.4	81.2	47.2	21.5	88.3
Use of bridges (%)^*^	18.2	15.3	21.7	35.8	38.5	32.8	22.4	27.5
Use of partial dentures (%)	5.6	7.7	11.4	21.5	43.6	58.2	43.0	22.9
Use of full dentures (%)	0.0	0.0	0.0	0.0	6.0	31.1	60.8	6.6
CPI score ≥3 (%)^†^	5.6	5.5	7.0	11.2	15.1	16.9	1.9	9.3
CPI score =3 (%)	5.6	4.0	6.3	8.1	13.0	13.9	1.9	7.6
CPI score =4 (%)	0.0	1.5	0.7	3.2	2.2	3.1	0.0	1.8
No index teeth for CPI measurement (%)	0.0	0.0	0.0	0.0	2.9	20.8	49.1	3.7
History of periodontal diseases with pockets (≥4 mm) (%)^*^	0.0	6.7	10.9	17.0	30.4	32.6	36.8	16.7
GOHAI score (mean ± SD)^‡^	56.0 ± 4.0	56.2 ± 4.7	55.8 ± 5.5	55.0 ± 5.3	54.1 ± 5.3	52.3 ± 7.7	52.7 ± 6.3	55.0 ± 5.7
(Number of participants with sufficient data)	(8)	(111)	(333)	(243)	(82)	(95)	(38)	(910)

^*^Surveyed in 31 of the 46 prefectural dental associations.

^†^CPI: Community Periodontal Index.

^‡^GOHAI: General Oral Health Assessment Index. Index was surveyed in 20 of the 46 prefectural dental associations.

### Validity of self-reported periodontal status

Of the 288 dentists who received the dental part of the questionnaire, 61 returned it and 50 (17.4% of the selected dentists) consented to undergo an oral examination. The mean age ± SD of the 50 participants was 53.3 ± 9.7 years (range, 33–73 years), and women accounted for 8.0% (*n* = 4) of participants. The mean interval ± SD between the reply to the questionnaire and the oral examination was 56 ± 36 days.

Table 4 shows the joint classification of CPI score as determined by oral examination and reported on the self-administered questionnaire. The consistency between the two sets of CPI scores was not high: the kappa coefficient was 0.53 (95% CI, 0.36–0.70). However, when the participants were dichotomized into those with or without a CPI score of ≥3 (Table 4), the kappa coefficient was 0.73 (95% CI, 0.53–0.93), with 87.5% sensitivity (95% CI, 61.7%–98.5%) and 88.2% specificity (95% CI, 72.6%–96.7%). Responses to the questionnaire indicated that periodontal status was assessed by a dental hygienist, another dentist, the respondent, or another person in 22 (44.0%), 14 (28.0%), 8 (16.0%), and 6 (12.0%) of the 50 participants, respectively. Even when the analysis was limited to assessments by the respondents themselves or another person, the kappa coefficient (0.85; 95% CI, 0.57–1.00) was not lower than that for assessments by a dental hygienist or another dentist (0.68; 95% CI, 0.43–0.93).

**Table 4.** (a) Joint classification of CPI score as assessed by oral examination and reported on self-administered questionnaire among 50 participants in a validation study. (b) Categorization of participants with or without a CPI score of ≥3.

**(a)**						

CPI score reported on questionnaire	CPI score assessed by oral examination					Total

	0	1	2	3	4	
0	8	0	2	1	0	11
1	4	4	0	0	0	8
2	4	0	8	1	0	13
3	3	0	1	10	2	16
4	0	0	0	0	2	2

Total	19	4	11	12	4	50

**(b)**			

CPI score reported on questionnaire	CPI score assessed by oral examination		Total

	0–2	≥3	
0–2	30	2	32
≥3	4	14	18

Total	34	16	50

## DISCUSSION

We have described the design of our nationwide prospective study of dentists and briefly presented the baseline lifestyle, clinical, and oral health characteristics of the participants. In addition, we validated the self-administered questionnaire on periodontal status by means of oral examinations. To our knowledge, this is the largest cohort study of dentists in Japan, a nation with relatively high mortality and incidence rates, among developed countries, for stroke and gastric cancer.^23^^,^^24^ Much attention has recently been paid to the relations between oral health and the risks of both stroke^25^ and gastric cancer^26^^,^^27^; information from the present study may increase our understanding of these relations.

To further characterize the cohort at baseline, we compared selected health-related characteristics of the cohort with those of the Japanese population by sex and age using data from available nationwide surveys, including The National Health and Nutrition Survey in Japan, 2004,^28^ The Survey of Dental Diseases, 2005 (data available on the Internet at http://www.mhlw.go.jp/topics/2007/01/tp0129-1.html), and the nationwide survey of GOHAI, which establishes national norms for Japanese (available at http://www.i-hope.jp/2007/09/qol_06.html) (5). In men in the present cohort, the proportion of current smokers was lower than in the general population, but the proportion of ex-smokers was higher. In particular, the percentage of male, current smokers younger than 60 years was more than 10 points lower than that of the general population. In women, however, smoking status among the cohort and general population was very similar, and the proportions of current drinkers were comparable, with the exception of middle-aged women. Obesity was similarly or less prevalent in the cohort, as compared with the general population, in middle-aged and elderly men and women. Hypertension was substantially more common in the younger age subgroups of the cohort, as compared with the general population.

**Appendix.** Selected health-related characteristics of the Japanese general population, by sex and age (summarized data from nationwide surveys)

	Age							
	
	20–29	30–39	40–49	50–59	60–69	70–79	≥80	
Men								
Current smokers (%)^*^	51.3	57.3	51.4	47.7	33.3	24.0		
Ex-smokers (%)^*^	6.9	11.3	19.3	27.2	32.2	38.1		
Current drinkers (%)^*,†^	64.6	72.0	75.8	76.5	73.0	57.2		
Ex-drinkers (%)^*^	0.5	1.1	2.1	0.6	2.3	4.9		
Body mass index ≥25.0 (%)^*^	19.9	28.9	32.7	30.8	29.7	25.5		
Hypertension (%)^*,‡^	7.3	16.4	44.0	53.7	67.7	72.9		
Brushing ≥3 times/day (%)^§^	9.4	9.0	10.7	10.8	15.2	18.4	18.7	
Tooth loss (mean number)^§,||^	0.2	0.5	2.0	4.6	8.1	14.2	18.4	
Number of present teeth ≥20 (%)^§^	100.0	99.4	95.9	85.2	65.3	37.9	24.7	
CPI score ≥3 (%)^§,¶^	20.3	29.4	36.3	53.8	55.5	46.6	34.4	
GOHAI score (mean)^**,††^	54.3	53.8	53.6	53.1	52.8	50.4	NA^‡‡^	

Women								
Current smokers (%)^*^	18.0	18.0	13.7	13.7	7.6	4.5		
Ex-smokers (%)^*^	5.2	7.8	6.0	2.7	3.8	4.0		
Current drinkers (%)^*,†^	51.0	49.6	47.2	38.3	29.4	17.9		
Ex-drinkers (%)^*^	1.9	2.8	1.0	0.5	1.4	1.7		
Body mass index ≥25.0 (%)^*^	5.4	8.3	17.9	24.1	29.9	26.7		
Hypertension (%)^*,‡^	1.1	6.5	21.9	38.8	55.9	75.2		
Brushing ≥3 times/day (%)^§^	30.8	28.9	27.1	26.0	27.5	26.8		17.9
Tooth loss (mean number)^§,||^	0.3	0.7	1.7	4.2	8.9	15.4		21.0
Number of present teeth ≥20 (%)^§^	100.0	99.6	96.7	85.0	61.7	34.4		12.7
CPI score ≥3 (%)^§,¶^	11.2	20.8	35.8	40.6	46.1	44.6		27.3
GOHAI score (mean)^**,††^	52.3	54.8	53.7	51.3	52.4	51.2		NA^‡‡^

^*^National Health and Nutrition Survey in Japan, 2004.

^†^Current drinkers were defined as individuals who consume alcohol at least once a month.

^‡^Systolic blood pressure ≥140 mm Hg, diastolic blood pressure ≥90 mm Hg, or taking medication for hypertension.

^§^Survey of Dental Diseases, 2005.

^||^Maximum number of teeth was adjusted to 28 for comparison with figures from present study.

^¶^CPI: Community Periodontal Index.

**GOHAI: General Oral Health Assessment Index.

^††^National norm for Japanese (mean).

^‡‡^NA: not available.

The cohort members generally had better oral health than the general population: they had a more diligent hygienic routine, fewer lost teeth, fewer periodontal diseases, and higher oral health-related QOL. In men, differences between the two populations in the mean number of teeth lost and the proportion of those with a sufficient number of teeth increased with age, but diminished in the very old. Thus, lifelong oral health was not easily attained, even among dentists with good oral hygiene.

Some methodological issues in the present study warrant consideration. First, the validity of self-reported oral health status needs to be confirmed. To address this concern, we conducted a validation study focusing on periodontal status that compared the responses on the questionnaire with the findings of dental examinations. The study demonstrated that the participants could be classified, with satisfactory validity, into those with or without periodontal pockets ≥4 mm in any of the index teeth used for CPI measurement (kappa coefficient, 0.73; sensitivity, 87.5% and specificity, 88.2%). The information collected with the self-administered questionnaire, therefore, should be useful when classifying respondents according to the presence of deep periodontal pockets. In the present baseline survey, periodontal status was not always assessed by a dental professional other than the participant: it was determined by a dental hygienist, another dentist, the respondent, or another person in 38.6%, 20.1%, 32.6%, and 8.6% of the participants, respectively. Although the respondents themselves and non-dental professionals cannot accurately measure CPI, they assessed periodontal status and classified it according to the criteria for periodontal status used for CPI scoring (score 0–4) in the present study. In terms of the assessment of periodontal condition, the validation study did not show lower validity for persons other than dental hygienists or other dentists when classifying individuals as those with or without a CPI score of ≥3. Nevertheless, in analyses of the cohort data, subgroup analyses that account for the profession of the assessors may prove informative.

Second, our cohort was comprised of oral health professionals, and findings from such a highly specific population cannot be straightforwardly generalized. Although the participants were in better oral health than the general population, there was still considerable inter-individual variation in the indices; some respondents scored poorly on the indices (eg, many lost teeth, high CPI score). Therefore, we expect to be able to compare subsequent mortality and morbidity between those with better and worse scores on indices. In addition, the cohort is relatively homogeneous in terms of socioeconomic status, so the effect of this potential confounder^29^^,^^30^—a concern in other studies^26^^,^^27^—will be minimized. Third, lack of direct measurement of blood pressure, blood lipids, and glucose levels may be a limitation when attempting to determine the association between oral health and cardiovascular disease. Fourth, we have not analyzed some potential occupational risks, such as infections and exposures to asbestos and mercury, because of the difficulty in doing so with a self-administered questionnaire comprising a limited number of pages. We therefore must consider the extent to which residual or unadjusted confounding by cardiovascular risk factors or occupational risk affects the association between oral health and systemic diseases.

Finally, because the response rate was not high (36.2%), the respondents are not a representative sample of JDA members. This fact should be kept in mind when interpreting the baseline profiles in this article. In addition, in generalizing the findings from this study to a designated target population, differences in the background characteristics of the cohort and the target population must be identified, and the relevance of such differences should be carefully considered from the perspective of the underlying biological mechanisms.^31^ Although dental status differed somewhat between the dentists and the general public, the relevant biological mechanisms are essentially identical among humans, and the associations between oral health and general health, as observed in dentists, should be applicable to the general population.

As of December 2007, the cohort members have been under observation for an average of 4 years. We expect the study to provide strong evidence for the link between oral health and general well-being.
